# Human S100A5 binds Ca^2+^ and Cu^2+^ independently

**DOI:** 10.1186/s13628-017-0040-y

**Published:** 2017-11-22

**Authors:** Lucas C. Wheeler, Michael J. Harms

**Affiliations:** 1 Department of Chemistry and Biochemistry, University of Oregon, Eugene, 97403 OR USA; 2 Insitute of Molecular Biology, University of Oregon, Eugene, 97403 OR USA

**Keywords:** S100A5, S100 proteins, ITC, AUC, Calcium binding proteins, Copper binding, Circular dichroism

## Abstract

**Background:**

S100A5 is a calcium binding protein found in a small subset of amniote tissues. Little is known about the biological roles of S100A5, but it may be involved in inflammation and olfactory signaling. Previous work indicated that S100A5 displays antagonism between binding of Ca^2+^ and Cu^2+^ ions—one of the most commonly cited features of the protein. We set out to characterize the interplay between Ca^2+^ and Cu^2+^ binding by S100A5 using isothermal titration calorimetry (ITC), circular dichroism spectroscopy (CD), and analytical ultracentrifugation (AUC).

**Results:**

We found that human S100A5 is capable of binding both Cu^2+^ and Ca^2+^ ions simultaneously. The wildtype protein was extremely aggregation-prone in the presence of Cu^2+^ and Ca^2+^. A Cys-free version of S100A5, however, was not prone to precipitation or oligomerization. Mutation of the cysteines does not disrupt the binding of either Ca^2+^ or Cu^2+^ to S100A5. In the Cys-free background, we measured Ca^2+^ and Cu^2+^ binding in the presence and absence of the other metal using ITC. Saturating concentrations of Ca^2+^ or Cu^2+^ do not disrupt the binding of one another. Ca^2+^ and Cu^2+^ binding induce structural changes in S100A5, which are measurable using CD spectroscopy. We show via sedimentation velocity AUC that the wildtype protein is prone to the formation of soluble oligomers, which are not present in Cys-free samples.

**Conclusions:**

S100A5 can bind Ca^2+^ and Cu^2+^ ions simultaneously and independently. This observation is in direct contrast to previously-reported antagonism between binding of Cu^2+^ and Ca^2+^ ions. The previous result is likely due to metal-dependent aggregation. Little is known about the biology of S100A5, so an accurate understanding of the biochemistry is necessary to make informed biological hypotheses. Our observations suggest the possibility of independent biological functions for Cu^2+^ and Ca^2+^ binding by S100A5.

**Electronic supplementary material:**

The online version of this article (doi:10.1186/s13628-017-0040-y) contains supplementary material, which is available to authorized users.

## Background

S100A5 is a member of the calcium-binding S100 protein family. The protein is primarily homodimeric and is capable of binding one Ca^2+^ ion each at it’s EF-hand and pseudo-EF-hand sites [1, 2]. S100A5 undergoes a notable conformational change upon calcium-binding, resulting in the rotation and extension of a helix [1]. This Ca^2+^-driven exposure of a hydrophobic surface is the primary mode of signal transduction in the S100 proteins [3]. Through interactions with metals and protein targets, S100s play a variety of biological roles including control of cell proliferation, inflammatory signalling, and antimicrobial activity [4–7].

S100A5 is expressed primarily in the olfactory bulb and olfactory sensory neurons (OSNs). Its expression is dramatically upregulated by odor stimulation [8–10]. It has been proposed that S100A5 is actively involved in olfactory signalling due to its expression profile [9]. Expression of the protein has also been observed in a small number of other tissues [10]. It is used as a bio-marker for several types of brain cancers and inflammatory disorders and appears to be involved in inflammation via activation of RAGE [2, 11, 12]. Genetic work on S100A5 has been minimal, which has limited our understanding of its biological roles.

The first biochemical study of human S100A5 identified it as a novel Ca^2+^, Cu^2+^, and Zn^2+^ binding protein [9]. The authors used flow-dialysis to measure binding of the metal ions to the protein and concluded that S100A5 is capable of binding four Ca^2+^ ions, four Cu^2+^ ions, and two Zn^2+^ ions per homodimer. One of the most striking observations of that study was the strong antagonism between the binding of Cu^2+^ and Ca^2+^ ions to the protein. This feature is one of the most highly cited aspects of S100A5. Because little is known about the protein, this fact is present in descriptions found across databases such as Uniprot, NCBI, Wikigenes, and Genecards [13–15]. While most S100s are capable of binding transition metal ions, antagonism with binding of Ca^2+^ is not known outside the S100A5 lineage. Thus, this unique feature of S100A5 provoked speculation about its possible biological implications [9, 16]. It was suggested that S100A5 might act as a Cu^2+^ and Ca^2+^ regulated signal during olfaction or as a Cu^2+^ sink to accommodate high Cu^2+^ concentrations in the olfactory bulb [9].

We sought to characterize this presumably important feature of S100A5 in more detail. Previously, we characterized the binding of Cu^2+^ and Zn^2+^ to a large number of S100 proteins including S100A5 [17]. Via ITC competition experiments, we established that these two metals bind at different sites on the protein and do not compete for binding [17]. We found that mutation of Cys43 and Cys79 lead to a loss of Zn^2+^ binding. In contrast neither of these residues was necessary for binding of Cu^2+^. Due to the original report of Ca^2+^ /Cu^2+^ antagonism we suspected that Ca^2+^ and Cu^2+^ may compete for the same sites on S100A5.

Here we report our study of the interplay between Ca^2+^ and Cu^2+^ binding by S100A5. Using a Cysteine-free variant (C43S/C79S) of the protein, we show that binding of Ca^2+^ and Cu^2+^ are not in fact antagonistic. The protein is capable of binding the two metals–which induce notable structural changes–simultaneously and independently. Furthermore, we establish that the Cysteine-containing (WT) protein is prone to the formation of high-ordered oligomers in solution, while the Cysteine-free variant is almost entirely dimeric. We suggest that this propensity for formation of large oligomeric species and precipitation under our experimental conditions may underlie the apparent antagonism observed in the original S100A5 report. Our results may suggest new biological roles for Cu^2+^ binding by this protein.

## Results

### Ca^2+^ and Cu^2+^ binding to S100A5 are not antagonistic

**Fig. 1:**
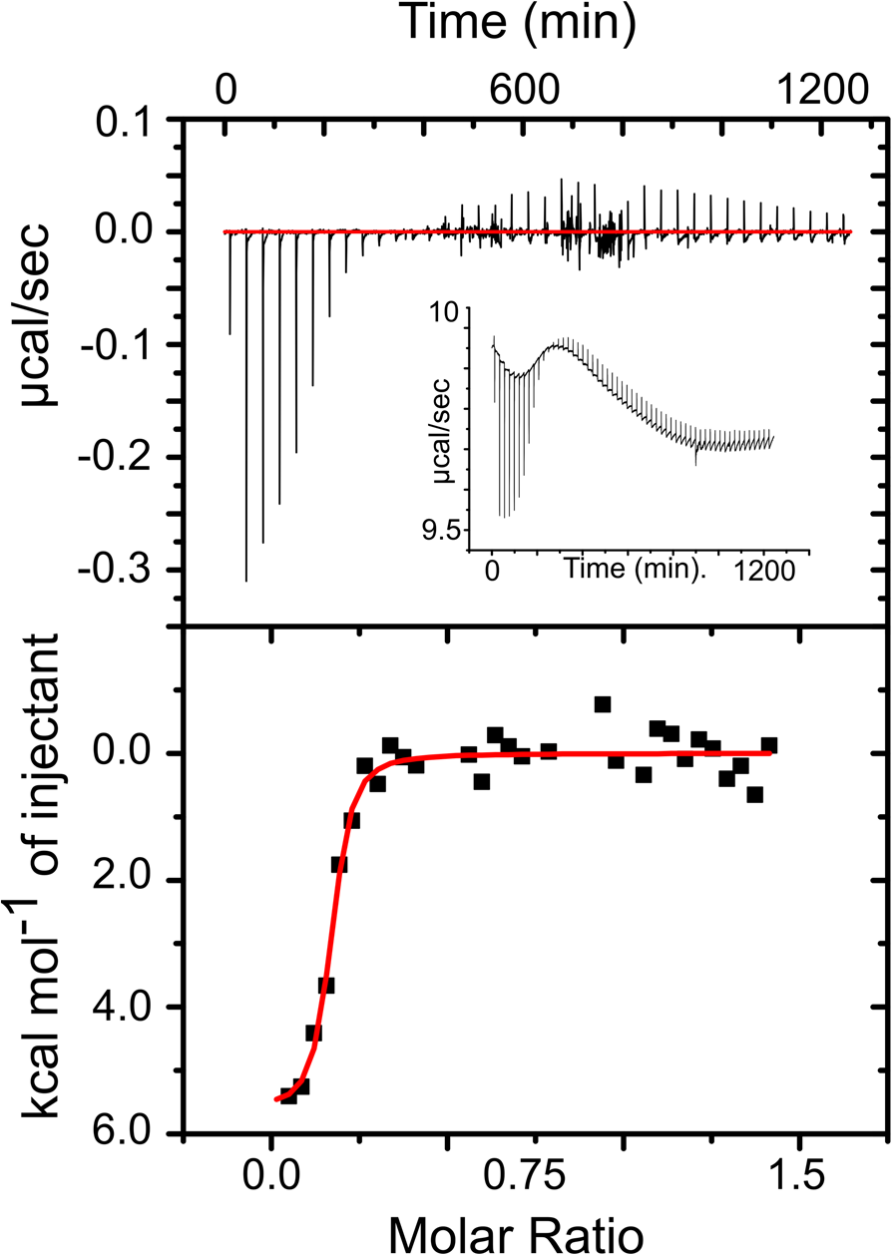
Measurements of Cu^2+^ binding to wildtype S100A5 in the presence of Ca^2+^ are difficult to interpret. Representative ITC trace showing Cu^2+^ titrated onto Ca^2+^-bound wildtype S100A5. Inset shows raw data trace. Data were characteristically noisy and the apparent fraction competent was systematically low

Antagonism between Cu^2+^ and Ca^2+^ binding was previously identified as a distinct feature of S100A5 relative to other S100 proteins [9, 16, 18]. We hypothesized that Cu^2+^ and Ca^2+^ may bind using the same ligands, thus explaining the antagonism as direct competition. It was suggested in the original paper that Cu^2+^ and Ca^2+^ might share some ligands [9]. We performed ITC competition experiments to test whether Cu^2+^ and Ca^2+^ directly compete. We titrated Cu^2+^ onto S100A5 in the presence of saturating Ca^2+^. However, these experiments were difficult to interpret due to extensive precipitation in the samples containing both ions. ITC traces were very noisy and apparent stoichiometries were systematically low (≈0.2), suggesting that a large portion of the protein sample was not competent to bind Cu^2+^ (Fig. 1). Together these observations suggested that a metal-driven aggregation process could be occurring in our samples.

We found previously that neither of the two native Cys residues in S100A5 were required for Cu^2+^ binding [17]. We also noticed that–unlike the wildtype protein–the Cys-free mutant did not precipitate in the presence of saturating Ca^2+^ and Cu^2+^. We thus sought to use ITC to characterize the interaction between binding of the two metal ions using the Cys-Ser double mutant. Because some of the metal-binding curves were complex and difficult to fit, we used a Bayesian Markov Chain Monte Carlo sampler–as implemented in pytc–to estimate thermodynamic parameters for all binding models [19]. We also included a floating “fraction competent” parameter to capture uncertainty in the relative protein and metal concentrations (following SEDPHAT [20]). This was necessary because a number of factors make it difficult to obtain accurate estimates of concentrations for components of this system. S100A5 has no tryptophan residues and, therefore, a low extinction coefficient that makes absorbance-based concentration estimates unreliable. Further, water absorption by dry metal salts, as well as interactions between metal ions and buffer, can also make estimates of metal concentration difficult. Because of these of uncertainties, ITC has been noted to provide poor estimates of stoichiometry for protein metal binding [21].

**Fig. 2:**
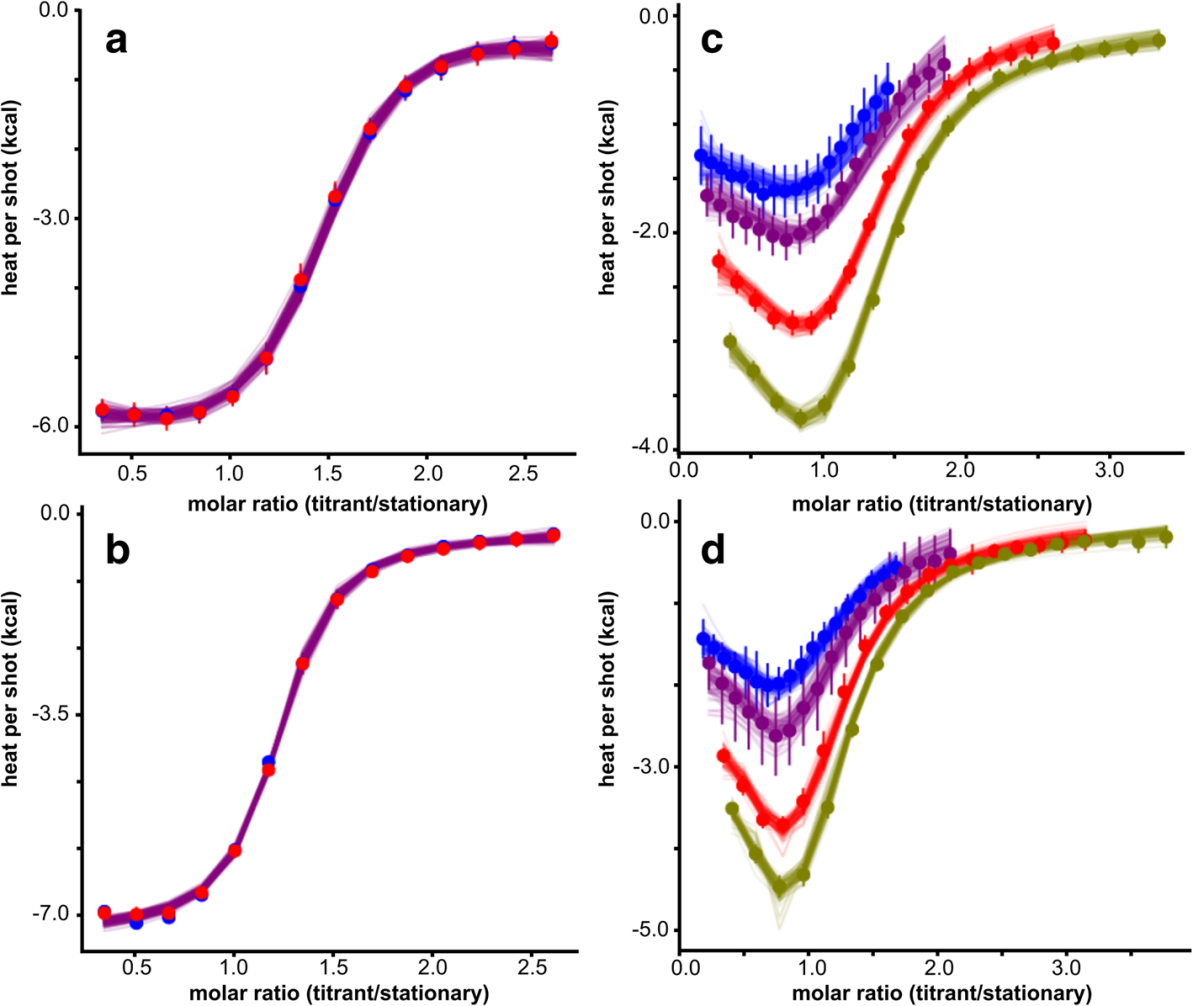
S100A5 can bind Ca^2+^ and Cu^2+^ simultaneously without antagonism. Plots show integrated data and global Bayesian fits from replicate isothermal titration calorimetry experiments: **(a)** Cu^2+^ binding to apo protein, (**b**) Cu^2+^ binding to Ca^2+^-saturated protein, (**c**) Ca^2+^ binding to apo protein, and (**d**) Ca^2+^ binding to Cu^2+^-saturated protein. Points are integrated titration shots. Lines are 100 curves drawn from the posterior distribution of the MCMC samples. For Cu^2+^ binding experiments technical replicates are shown in blue and red. Ca^2+^ binding experiments were performed with fixed protein concentration and four different titrant/titrate ratios: 8X (blue), 10X (purple), 15X (red), and 18X (green). For clarity Y-axes display total heat per shot, so that curves from different titrant concentrations fall on different areas of the graph. Raw data corresponding to these integrated heats are displayed in Additional file 1: Figure S1

We first used ITC to remeasure binding of Cu^2+^ ions to the apo form of the S100A5 double mutant. We found the Cu^2+^ binding data was best described with a single-site binding model. In line with our previous observations, the protein bound Cu^2+^ with a *K*_*d*_(*μ* *M*) that had a 95% credibility region of 0.94≤1.81≤3.90 (Fig. 2 a, Table 1). We next measured the binding of Cu^2+^ in the presence of saturating Ca^2+^. Ca^2+^ had no detectable effect on the binding of Cu^2+^ to the protein, giving a *K*_*d*_(*μ* *M*) of 0.65≤0.96≤1.47 (Fig. 2 b; Table 1).

**Table 1:** Fit parameters from pytc fits

Ion	*Cu* ^2+^		*Ca* ^2+^	
Competitor	None	*Ca* ^2+^	None	*Cu* ^2+^
*Δ* *H*_1_	− 5.7≤−3.4≤−2.8	− 4.1≤−3.6≤−3.2	− 1.8≤−1.4≤−0.7	− 1.5≤−1.2≤−0.7
*Δ* *H*_2_	–	–	− 5.7≤−4.6≤−3.7	− 5.0≤−4.1≤−3.4
*K*_*d*,1_	0.9≤1.8≤3.9	0.7≤1.0≤1.5	0.4≤0.5≤2.7	0.03≤0.2≤2.2
*K*_*d*,2_	–	–	1.9≤6.3≤34.9	1.9≤10.5≤100
fx. comp.	1.40≤1.43≤1.47	1.15≤1.17≤1.19	0.61≤0.66≤0.69	0.54≤0.58≤0.61

Table contains values for key parameters determined via global fits of ITC data using the Bayesian MCMC fitter in pytc. Ninety-five percent credibility regions from the posterior distributions are reported for parameter values. *Δ* *H* values are reported in *kcal*·*mol* ^−1^, *K*_*d*_ values in *μ* *M*. Final parameter is fraction competent, a nuisance parameter that captures what fraction of the metal and protein in solution are competent for the measured reaction

We next performed the inverse set of experiments. We used ITC to measure the binding of Ca^2+^ to the protein in the apo and Cu^2+^ –saturated forms. For each condition, we used four different titrant/stationary ratios to better resolve the complex Ca^2+^ binding curve and then globally fit a binding model to all four datasets (Fig. 2 c). This binding curve had two distinct phases and could be fit with a two-site binding polynomial (Fig. 2 c). These Ca^2+^ binding curves presented a challenging model-fitting problem due to the complex shape of the curve. The individual enthalpies and binding constants may therefore be under-determined in our analysis. To resolve realistic parameter values from the binding polynomial model, we constrained the dilution heat and dilution intercept in the Bayesian fit to reasonable values.

We observed one high-affinity site (*K*_*d*_(*μ* *M*): 0.14≤0.46≤2.68) and one lower-affinity site (*K*_*d*_(*μ* *M*): 1.85≤6.33≤34.88). The values were roughly consistent with those reported in the literature [2]. The presence of saturating Cu^2+^ did not inhibit the binding of Ca^2+^ ions (Fig. 2 d; Table 1). The *K*_*d*_ value of the low affinity site (*K*_*d*_(*μ* *M*): 0.03≤0.18≤2.16) was not distinguishable within uncertainty from that of the apo protein. The *K*_*d*_ of the high affinity site (*K*_*d*_(*μ* *M*): 1.86≤10.46≤100.3) is similarly indistinguishable from that for the apo protein (Table 1). Our results clearly demonstrate that Ca^2+^ and Cu^2+^ ions do not display strong antagonism when binding to S100A5.

### S100A5 is prone to oligomerization and metal-driven aggregation

We hypothesized that the metal-driven aggregation process observed in our ITC experiments with the wildtype protein contributed to the apparent antagonism that was previously reported. To further examine this aggregation process we used sedimentation velocity AUC to test for the presence of oligomers in solution. We hypothesized that the oligomerization of the wildtype protein was driven by the presence of Cysteine residues. Due to the presence of Cu^2+^ in some samples we were unable to use a reducing agent in either the ITC or AUC experiments.

**Fig. 3:**
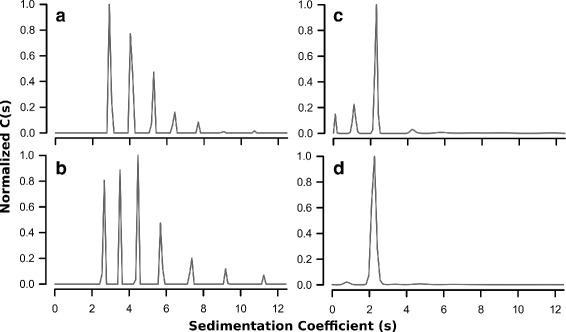
Wildtype S100A5 is prone to the formation of high-ordered oligomers. Sedimentation velocity AUC distribution plots showing (**a**) apo wildtype S100A5, (**b**) wildtype S100A5 saturated with Cu^2+^ and Ca^2+^, (**c**) apo Cys-Ser double mutant, and (**d**) Cys-Ser double mutant saturated with Cu^2+^ and Ca^2+^. Data are normalized to the same scale. Homodimers are the peaks near *s*=2. The Cys-Ser double mutant plots show evidence of some monomer (peak near *s*=1) in solution

We performed sedimentation velocity AUC experiments on both the wildtype and Cys-Ser double mutant proteins in both the apo form and the form loaded simultaneously with Cu^2+^ and Ca^2+^. We fit the Lamm equation to the sedimentation data using SedFit to calculate the c(s) distribution of the protein in each condition [22, 23]. We found that apo S100A5 formed high-ordered oligomers, ranging to at least dodecamers (Fig. 3 a). Addition of Cu^2+^ and Ca^2+^ caused a large amount of precipitation in wildtype S100A5 that we removed by extensive centrifugation prior to loading the cell. The remaining soluble protein was indistinguishable from the apo protein (Fig. 3 b). In contrast, when we performed the same experiments with the Cys-Ser double mutant we found that the protein was primarily dimeric in solution (Fig. 3 c, d), even with the addition of Cu^2+^ and Ca^2+^. However, monomers were also detectable in the double mutant samples. The monomer peak appears to be more prominent in the apo-protein sample than in the sample saturated with Cu^2+^ and Ca^2+^, suggesting that binding of metals may stabilize the dimeric form (Fig. 3 c, d). Our AUC results clearly demonstrate that oligomerization of S100A5 is driven by the native cysteine residues, which also likely cause the visible aggregation we observed in the ITC experiments. This observation strongly suggests that the previously-reported apparent antagonism between Ca^2+^ and Cu^2+^ was due to oligomerization and/or aggregation.

### Binding of Ca^2+^ and Cu^2+^ induce reversible changes in S100A5 secondary structure

**Fig. 4:**
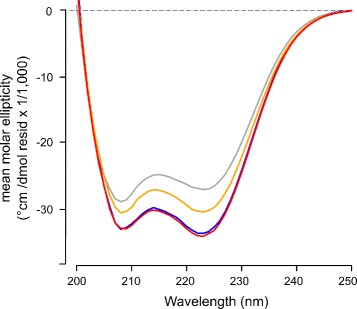
Ca^2+^ and Cu^2+^ induce increases in *α*-helical secondary structure measured by far UV circular dichroism. Curves show mean molar ellipticity vs. wavelength for each experimental condition: Apo (gray), bound to Cu^2+^ (orange), bound to Ca^2+^ (blue), and bound to both Cu^2+^ and Ca^2+^ (red)

One hallmark feature of the S100 proteins is the change in secondary structure observed upon binding of metal ions [17, 24, 25]. Metal-induced conformational changes expose a binding interface that can bind downstream targets and regulate their activities [2, 3]. In the original publication on S100A5 biochemical characterization, the authors found that the secondary structure of the protein is insensitive to the binding of metal ions [9]. However, we previously found that binding of Ca^2+^ ions to wildtype S100A5 induces a significant (≈25%) reversible increase in *α*-helical secondary structure, which is consistent with the changes observed in published NMR data [1, 2]. Due to instantaneous sample precipitation, we were unable to reliably measure structural changes of wildtype S100A5 in the presence of Cu^2+^. However, the Cys-Ser double mutant protein alleviates this issue. We collected far-UV circular dichroism spectra of the mutant protein in the apo form and bound to Ca^2+^, Cu^2+^, and Ca^2+^ and Cu^2+^ simultaneously. The Cys-Ser mutant displays a notable increase in alpha-helical signal (222 nm) upon binding of Ca^2+^, identical to the wildtype protein. Interestingly, addition of Cu^2+^ also induces an increase in *α*-helical signal that is approximately half of that induced by Ca^2+^. The spectrum of S100A5 bound simultaneously to both metals is identical to that of the Ca^2+^-bound form (Fig. 4). This structural change is not due to oligomerization, as the protein remains a dimer under these conditions by AUC (Fig. 3 d). All the metal induced structural changes were instantly reversible by the addition of a molar excess of EDTA. These results may help to explain the minor differences–such as larger enthalpy–in Ca^2+^ binding to the Cu^2+^ –bound form of the protein, which may be due to moderate structural differences from the apo-protein. Despite the lack of antagonism between binding affinities for Ca^2+^ and Cu^2+^ ions, there is still indication of some structural interplay between the two metals.

## Discussion

S100A5 is one of the lesser-known members of the S100 protein family. Its expression pattern is very narrow and its biological functions are mostly uncharacterized. However, it has been the target of multiple biochemical studies that have sought to characterize the properties of the protein itself. Binding of metals and proteins to S100A5 have been studied using various techniques [1, 2, 9, 11, 17]. X-ray crystallography and NMR have been used to solve structures of both apo and Ca^2+^ –bound forms of the protein [1, 26]. Despite the available biochemical data aspects of S100A5 have remained ambiguous. For example, the stoichiometry of transition metal binding and structural responses to metal binding have been variably reported [9, 17].

One of the most noted features of S100A5 is the strong antagonism between binding of Ca^2+^ and Cu^2+^ ions. This feature is reported in the gene descriptions found in many databases [13–15]. In this study we set out to characterize this unique feature of S100A5, hypothesizing that it was due to competition between the two metals for shared ligands. However, we found an absence of direct binding antagonism between Ca^2+^ and Cu^2+^. Neither metal ion affects the binding constant for the other. Instead, we observed a propensity of the protein for oligomerization and metal-induced aggregation. It is possible that the reduction of binding-competent protein caused by this aggregation process was interpreted in the original flow dialysis study of S100A5 as antagonism between Ca^2+^ and Cu^2+^. We also report notable changes in the secondary structure of S100A5 upon binding of both Ca^2+^ and Cu^2+^, which is contrary the original report that S100A5 structure is insensitive to the binding of metals.

One intriguing implication of our observations is that the Cu^2+^ binding site of S100A5 must be quite distinct from that of other S100 proteins. Ca^2+^ and Cu^2+^ clearly do not share ligands, or there would be evidence of competition in our ITC experiments. Cysteine residues are thought to be involved in metal-binding in some other S100s [16, 27] and we previously showed that the Cys-free mutant of S100A5 displays compromised Zn^2+^ binding [17]. However, neither native Cys residue of S100A5 is required for Cu^2+^ binding. Furthermore, we showed that Zn^2+^ and Cu^2+^ do not share ligands, as they do not compete at all in ITC experiments [17]. In addition, mutation of His17–which is present in the canonical transition metal site of many S100s–also had no effect on Cu^2+^ binding in S100A5 [17]. The results presented here with the Cys-free mutant also clearly rule out the possibility of oligomer-dependent Cu^2+^ binding, such as could be achieved by the formation of a new site in a high-order oligomeric species. Thus, we still have no clues as to where Cu^2+^ ions bind on S100A5. Further characterization–such as via scanning mutagenesis–will be necessary to determine the identity of Cu^2+^ ligands.

Biological roles for the binding of transition metals have been established for some S100s and suggested for many others [16, 18, 27–29]. The binding constants that we measured for Ca^2+^ and Cu^2+^ suggest the possibility of physiologically relevant interactions in some tissues. Free Ca^2+^ concentrations in rat olfactory neurons reach ≈2 *μ* *M* during nerve stimulation [30]. Likewise, pools of Cu^2+^ are released in and around olfactory neurons during signaling, reaching concentrations as high as 10 *μ* *M* in the synapse [31–34]. Further, despite high Cu^2+^ concentrations, the olfactory bulb in rats does not have elevated expression of the typical copper chaperone metallothionein [35]. It has been suggested that S100A5 may play a role as a Cu^2+^ buffer or chaperone in OSNs during olfactory signaling [9]. The fact that Cu^2+^ is able to induce structural changes in S100A5 suggests it could play a more active role: S100A5 could actually respond to Cu^2+^ and propagate a resulting signal by interacting with downstream targets.

Due to lack of antagonism, Cu^2+^ –dependent functions could be achieved even in the presence of saturating Ca^2+^ levels. Furthermore, there could be synergistic functional roles for binding of Ca^2+^ and Cu^2+^. For example, if S100A5 is acting as a Cu^2+^ chaperone, binding of Ca^2+^ could facilitate binding of protein targets–via exposure of the hydrophobic binding interface–to which Cu^2+^ is being delivered. Furthermore, S100A5 is capable of binding Zn^2+^ ions–which are also at high concentration in the olfactory bulb–with similar affinity to Cu^2+^ [34]. Zn^2+^ and Cu^2+^ also bind noncompetitively and thus all three metals could potentially engage in synergistic activities [17].

One final possibility is that the oligomerization process we observed in this study may actually have a biological function. Wildtype S100A5 is prone to the formation of oligomers even in the apo form and is subject to extensive aggregation in solutions containing Ca^2+^ and Cu^2+^ even at relatively low protein concentrations. Roles for metal-driven oligomerization in S100s have been suggested previously [7, 36–38]. It is conceivable that Ca^2+^ and Cu^2+^ drive oligomerization of S100A5 in cells to facilitate a biological function, but further experiments would be required to determine if this process occurs in the reducing environment of the cell at physiologically-relevant concentrations of S100A5, Ca^2+^ and Cu^2+^.

Future experiments are needed to elucidate the biochemical features and biological functions of S100A5 that remain unknown. It will be important to identify the Cu^2+^ ligands in S100A5 to fully understand the biochemical interplay between the binding of various biologically relevent metals. To understand how Ca^2+^, Cu^2+^, and Zn^2+^ contribute to the biological activity of S100A5, experiments should be targeted at directly testing how these metals interact with the protein in vivo. The identification of more S100A5 biological targets and an increase in functional studies will be required to determine the chief roles of S100A5 in its cellular environment.

## Conclusions

Antagonism between binding of Ca^2+^ and Cu^2+^ ions to S100A5 is one of the most oft-cited aspects of this protein. Several possible biological roles have been suggested. Using careful biophysical characterization, we discovered that binding of Ca^2+^ and Cu^2+^ ions is not antagonistic. A Cys-free mutant version of the protein makes measurements of metal binding using ITC possible and shows that the protein is capable of binding both metals simultaneously and independently. Rather than binding antagonism, it appears that the wildtype protein is prone to oligomerization and aggregation and that these behaviors may have contributed to the original interpretation. Furthermore, we also measured the effects of Ca^2+^ and Cu^2+^ binding on S100A5 secondary structure and found that both metals are capable of inducing increases in *α*-helical secondary character. These results also contrast the original report on S100A5 [9], but are consistent with previously published NMR data [1]. The ability to bind Ca^2+^ and Cu^2+^ independently as well as the structural response to Cu^2+^ may suggest new Cu^2+^ –dependent biological roles for S100A5.

## Methods

### Protein expression and purification

We previously generated the 6-histidine-tagged cysteine double-mutant construct in a pet28/30 vector [17]. In this study, the protein was expressed and purified using the same protocol detailed in the previous publication. Briefly, the protein was expressed in a 1.5L culture of Rosetta (DE3) pLysS cells (Millipore). Cells were lysed by sonication and treatment with DNase and lysozyme. Subsequently, the tagged protein was purified using HisTrap Ni^2+^ affinity columns (GE). The tag was then cleaved using TEV protease and the cleaved protein was further purified using Ca^2+^-dependent hydrophobic interaction chromatography. Finally, the sample was run over a second HisTrap Ni^2+^ affinity column to remove any uncleaved protein. The purified protein was dialyzed with 6000-8000 MWCO tubing (Fisher) against 2L 25 mM Tris, 100 mM NaCl, pH 7.4 with 2g chelex resin (BioRad). The dialyzed protein was filter-sterilized (0.22 *μ* *m*), flash-frozen dropwise in liquid nitrogen, and stored at − 80°*C*. We experimentally determined the extinction coefficient (5002*M* ^−1^ *cm* ^−1^) of the Cys-Ser double mutant. We measured the *A*_280_ of the protein at the same concentration in both buffer and denaturing 6M GdHCl (Sigma). We used ProtParam [39] to predict an extinction coefficient for the protein based on sequence and then calculated the corrected coefficient using the equation *ε*_*native*_=*ε*_6*MGdm*_·*A*_280,*native*_/*A*_280,6*MGdm*_. Concentration measurements were also corrected for scattering in samples [40]. Due to the low extinction coefficient of the protein, concentration is difficult to measure with high confidence, even with this careful protocol.

### Isothermal titration calorimetry

Samples were prepared in 25 mM TES (Sigma), 100 mM NaCl (Thermo Scientific), buffer at pH 7.4. Protein was thawed from the frozen stock and exchanged into the experimental buffer using NAP-25 desalting columns (GE Healthcare). For competition experiments the experimental buffer also contained either 1 mM *CaCl*_2_ (Sigma) or 0.25 mM *CuCl*_2_ (Sigma). Titrant solutions were prepared in matching experimental buffer to ensure identical conditions to titrate. Anhydrous *CaCl*_2_ or *CuCl*_2_ was dissolved directly in the buffer and diluted to the appropriate concentration immediately prior to experiments. Fresh stocks were made for each set of experiments. Experiments were performed with 50-80 *μ* *M* protein at 25 °C. Two technical replicates of each Cu^2+^ binding experiment were performed. To resolve the complex Ca^2+^ binding curves, four Ca^2+^ binding experiments were performed using four different concentrations of titrant. Raw data were integrated using the NITPIC software package–which allows uncertainty in the baseline–and the integrated heats were exported in standard SedPhat format [41]. We then used the Bayesian MCMC iterator included in pytc to estimate model parameters against all experiments simultaneously [19]. We used the maximum likelihood estimate as a starting point and then explored the likelihood surface with 100 walkers, each taking 20,000 steps. We discarded the first 10% of steps as burn in. We restricted parameters against all experiments simultaneously. We verified convergence by performing the sampling procedure several times. A single site binding model was used for Cu^2+^ titration data and a two-site binding polynomial was used for Ca^2+^ titration data [42, 43]. For Ca^2+^ binding fits, we constrained the dilution heat and dilution intercept to between -3.0–0.0 kcal/mol and 0–10,000 kcal/mol/shot respectively. All other priors were uniform.

### Sedimentation velocity analytical ultracentrifugation

Experiments were done in 25 mM TES (Sigma), 100 mM NaCl (Thermo Scientific), 100 *μ* *M* EDTA at pH 7.4 with the appropriate metal added directly to the buffer during preparation. Metals were added to a final concentration of 250 *μ* *M*. Samples were prepared at 40 *μ* *M* in the appropriate experimental buffer by overnight dialysis (6000-8000 MWCO) against 2L at 4 °C. Before ultracentrifugation samples were centrifuged at 18,000×*g* at 4 °C in a temperature-controlled centrifuge for 30 min. Ultracentrifugation was done with sapphire windows at 50,000×*g* in sector-shaped cells (Beckman) on a Beckman ProteomeLab XL-1. Sedimentation was monitored using interference mode rather than absorbance at 280 nm due to the low extinction coefficient of S100A5. The Lamm equation was fit to the sedimentation data–using SedFit–to calculate the continuous c(s) distribution [22, 23]. Estimated sedimentation coefficients of the species present in solution were calculated from the numerical fits.

### Circular dichroism spectroscopy

Far-UV circular dichroism spectra (200–250 nm) were collected on a J-815 CD spectrometer (Jasco) with a 1 mm quartz cell (Starna Cells, Inc.). We prepared 50 *μ* *M* samples in a Chelex (Bio-Rad) treated, 25 mM TES (Sigma), 100 mM NaCl (Thermo Scientific), 100 *μ* *M* EDTA, buffer at pH 7.4. Samples were subsequently diluted to 25 *μ* *M* in buffers containing: no metal (apo), 1 mM Ca^2+^, 1 mM Cu^2+^, or both 1 mM Ca^2+^ and 1 mM Cu^2+^ –all prepared in the stock buffer above. Samples were centrifuged at 18,000×*g* at 25 °C in a temperature-controlled centrifuge (Eppendorf) before experiments. Spectra were collected at 25 °C in a Jasco peltier multi-cell sample unit. Reversibility of metal-induced structural changes was confirmed by adding a molar excess of EDTA to the metal-saturated samples and repeating spectra collection. In all cases, addition of EDTA returned the samples to the apo state. Five scans of each condition were collected. These scans were then averaged–using Jasco spectra analysis software–to minimize noise. Buffer blank spectra were generated for each condition. Applicable blanks were subtracted in the Jasco spectra analysis software. Blank-corrected data were exported as text files and raw signal was converted into mean molar ellipticity using the concentration and the number of residues (*N*_*res*_=95) in our S100A5 construct using the equation: *MME*=*CD*_*signal*_/*c*(*M*)·10·*L*(*cm*)·*N*_*res*_.

## Additional file

Additional file 1
**Figure S1** — Raw data corresponding to integrated heats and global fits in figure 2 of main text. a) hA5 binding Cu^2+^, b) Ca^2+^ – loaded hA5 binding Cu^2+^, c) hA5 binding Ca^2+^, and d) Cu^2+^ –loaded hA5 binding Ca^2+^. (TIFF 2673 kb)
