# Supplementary figures and images for: Human S100A5 binds Ca2+ and Cu2+ independently
Source: BMC Biophys. 2017 Nov 22;10:8. doi: 10.1186/s13628-017-0040-y (PMC5700546; doi:10.1186/s13628-017-0040-y)

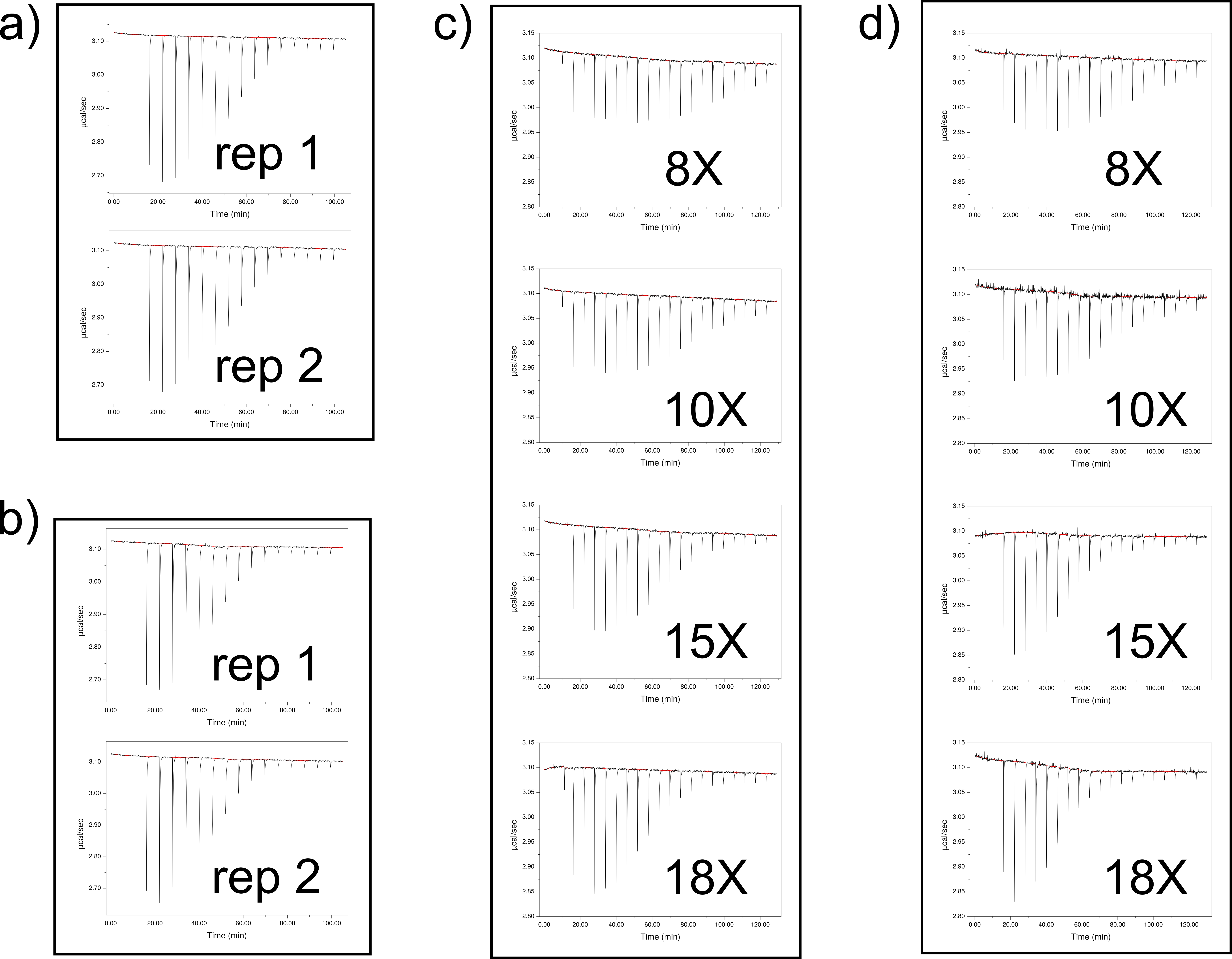

Supplement: Additional file 1 — Figure S1 — Raw data corresponding to integrated heats and global fits in figure 2 of main text. a) hA5 binding Cu2+, b) Ca2+ – loaded hA5 binding Cu2+, c) hA5 binding Ca2+, and d) Cu2+ –loaded hA5 binding Ca2+. (TIFF 2673 kb) [file 13628_2017_40_MOESM1_ESM.tiff]
